# Decitabine in combination with fludarabine and cyclophosphamide as a lymphodepletion regimen followed by CD19/CD22 bispecific targeted CAR T-cell therapy significantly improves survival in relapsed/refractory B-ALL patients

**DOI:** 10.1186/s40164-023-00397-z

**Published:** 2023-04-10

**Authors:** Yunju Ma, Haiping Dai, Qingya Cui, Sining Liu, Liqing Kang, Xiaying Lian, Wei Cui, Jia Yin, Lingling Liu, Mengjie Cai, Lei Yu, Depei Wu, Xiaowen Tang

**Affiliations:** 1 National Clinical Research Center for Hematologic Diseases, Jiangsu Institute of Hematology, The First Affiliated Hospital of Soochow University, Suzhou, China; 2 Institute of Blood and Marrow Transplantation, Collaborative Innovation Center of Hematology, Soochow University, Suzhou, China; 3 Shanghai Unicar-Therapy Bio-Medicine Technology Co.Ltd, Shanghai, China

**Keywords:** Decitabine, Chimeric antigen receptor T cell, Relapsed/refractory, Acute lymphoblastic leukemia, Lymphodepletion

## Abstract

Relapse is a major limitation of chimeric antigen receptor (CAR) T-cell therapy. Here, we speculated that decitabine (DAC) in combination with fludarabine and cyclophosphamide (FC) as a lymphodepletion regimen may improve the efficacy of CD19/CD22 CAR T-cell therapy. Fourteen of 26 patients with relapsed/refractory B cell acute lymphoblastic leukemia (r/r B-ALL) without remission before lymphodepletion treatment were treated with DAC (total dose 100 mg/m^2^ in 3 days) followed by the FC regimen (DAC group), while twelve patients received the FC regimen (CON group). On Day 28 after CAR T-cells infusion, no significant differences in complete remission (CR) and minimal residual disease negative CR rates were found between both groups. However, there were significant differences in overall survival (OS) and leukemia-free survival (LFS) between two groups: 3-year OS, 92.3% (DAC) versus 41.7% (CON), P = 0.005 and 3-year LFS, 92.9% (DAC) versus 27.3% (CON), P < 0.001. There was no significant difference in the incidence of cytokine release syndrome between both groups. Median time to platelet and neutrophil counts recovery was similar in both groups. All adverse events were reversible and manageable. In conclusion, DAC in combination with the FC lymphodepletion regimen may be a new treatment option that can improve the efficacy of CAR T-cell therapy in r/r B-ALL.

**Supplementary Information:**

The online version contains supplementary material available at 10.1186/s40164-023-00397-z.

**To the editor**

Antigen escape-mediated relapse is a major limitation of chimeric antigen receptor (CAR) T-cell therapy. One strategy to overcome antigen escape following CAR T-cell therapy is to generate T cells simultaneously targeting both CD19 and CD22. Although some clinical trials, using CD19/CD22 bispecific CAR T-cell therapy, have demonstrated promising therapeutic efficacy. CD19/CD22 CAR T-cell therapy has not induced durable remissions or reduced the relapse rate in patients with relapsed/refractory B-cell acute lymphoblastic leukemia (r/r B-ALL) [1, 2]. Relapse after CD19/CD22 CAR T-cell therapy is often associated with poor persistence of CAR T- cells in part caused by T cell exhaustion and the immunosuppressive microenvironment, which suggests the need for novel strategies to improve CD19/CD22 CAR T-cell therapy.

The hypomethylating agent decitabine (DAC) has been demonstrated to reverse T cell exhaustion, increase antigen expression, enhance T cell activation and modify the tumor microenvironment [3–7]. In addition, DAC combined with cytotoxic chemotherapy represents a promising strategy for the treatment of patients with high tumor burden [8], which is a significant predictor of poor prognosis in B-ALL. Therefore, we speculated that DAC in combination with fludarabine and cyclophosphamide (FC) as a lymphodepletion regimen may synergize and improve the efficacy of CAR T-cell therapy.

We retrospectively analyzed 26 r/r B-ALL patients without remission before lymphodepletion treatment who were enrolled in a phase 1/2 clinical trial of CD19/CD22 CAR T-cell therapy (NCT03614858) from October 2017 to May 2021 at the First Affiliated Hospital of Soochow University (Additional file 1: Figure S1). Fourteen patients received DAC combined with the FC regimen (DAC group) while twelve patients were treated with FC alone (CON group) followed by CAR T-cell therapy. The patients received DAC combined with FC depending on disease characteristics such as TP53 mutation, comorbidities, patients' wishes, and economic burden. Patients received the following lymphodepletion regimen: FC (fludarabine 30 mg/m^2^/day and cyclophosphamide 300 mg/m^2^/day) on days -5 to -3, with or without DAC (total dose 100 mg/m^2^ from day -6 to -4; Additional file 2: Figure S2).

There were more patients (42.9%) who relapsed after allogeneic hematopoietic stem cell transplantation (allo-HSCT) prior to CAR T-cell therapy in the DAC group than in the CON group (P = 0.017) (Table 1). On Day 28 after CAR T-cells infusion, no significant difference in minimal residual disease negative CR rates was found between both groups (Additional file 3: Table S1). Among the nontransplant patients in the DAC group, only one patient (16.7%, 1/6) relapsed. However, 1 of four nontransplant patients in the CON group had no response after CAR T-cell therapy and 3 patients relapsed after CAR T-cell treatment (Fig. 1A).

**Table 1:** Baseline Characteristics of Patients

Baseline characteristics of patients			
Characteristic	DAC group (14)	Control group (12)	P value
Gender			
Male	10 (71.4%)	5 (41.7%)	0.233
Female	4 (28.6%)	7 (58.3%)	
Age, median	26.5 (8–52)	31 (16–74)	0.226
Ph + ALL, n (%)	2 (14.3%)	5 (41.7%)	0.190
Ph-like ALL, n (%)	3 (21.4%)	2 (16.7%)	1.000
White blood cell ≥ 50 × 10^9/L, n (%)	6 (42.9%)	2 (16.7%)	0.216
Extramedullary leukemia, n (%)	2 (14.3%)	0 (0%)	0.483
Monosomal karyotype, n (%)	0 (0%)	1 (8.3%)	0.462
Complex karyotype, n (%)	2 (14.3%)	2 (16.7%)	1.000
KMT2A rearranged, n (%)	1 (7.1%)	0 (0%)	1.000
TP53 mutation or deletion, n (%)	2 (14.3%)	0 (0%)	0.483
T315I mutation, n (%)	1 (7.1%)	2 (16.7%)	0.580
Poor-risk cytogenetics, n (%)	8 (57.1%)	8 (66.7%)	0.701
Prior cycles of therapy, median (range)	4 (2–16)	6.5 (1–20)	0.251
< 5 cycles of therapy, n (%)	9 (64.3%)	4 (33.3%)	0.235
≥ 5 and < 10 lines of therapy, n (%)	2 (14.3%)	5 (41.7%)	
≥ 10 cycles of therapy, n (%)	3 (21.4%)	3 (25%)	
Primary refractory to chemotherapy, n (%)	2 (14.3%)	3 (25%)	0.635
Numbers of relapses, median (range)	1 (0–2)	1 (0–2)	0.685
Relapse after previous HSCT, n (%)			
Yes	6 (42.9%)	0 (0%)	0.017
No	8 (57.1%)	12 (100%)	
Blasts in BM before lymphodepletion treatment, median (range)	35.75 (5–82)	43.75 (6–85.5)	0.487
LDH, pre-lymphodepletion, median (range)	173.8 (106.5–644)	168.7 (83.6–722.2)	0.956
Ferritin, pre-lymphodepletion, median (range)	774.945 (234.06–2718.84)	1614.66 (411.63–2663.69)	0.140
CRP, pre-lymphodepletion, median (range)	5.115 (0.28–15.36)	1.195 (0.12–15.36)	0.170
ECOG, pre-lymphodepletion, median (range)	2 (1–3)	2 (1–3)	0.609
CAR T-cell dose, 10^7/kg, median (range)	1 (0.5–2)	1 (0.5–2.5)	0.950
Source of CAR T-cells, n (%)			
Autologous	10 (71.4%)	12 (100%)	0.100
Donor-derived allogenic	4 (28.6%)	0 (0%)	
Transduction rate (%), median	31.435 (13.02–64.35)	44.685 (6.07–63.92)	0.643

**Fig. 1:**
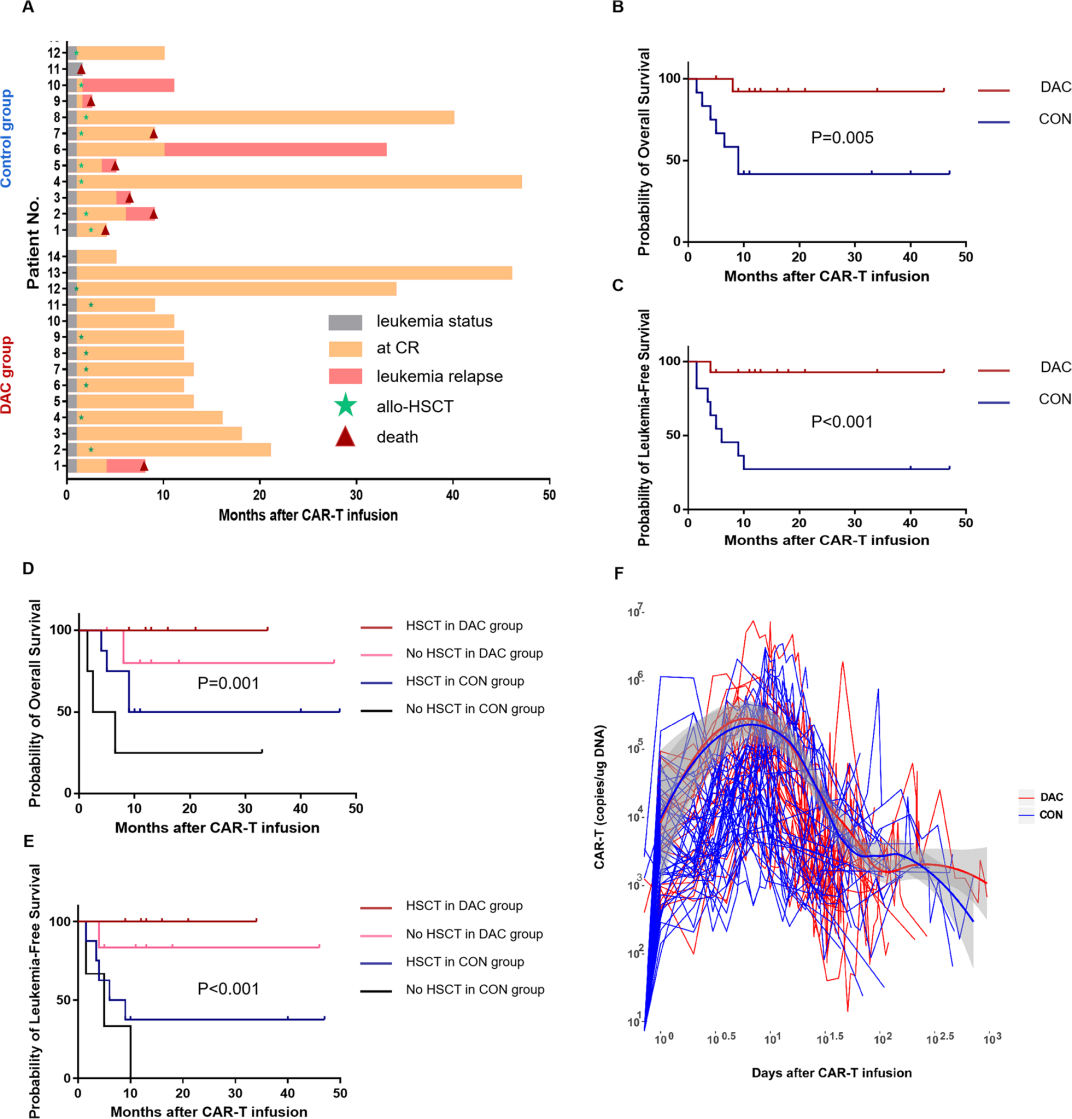
Treatment response, overall survival probability, long-term prognosis and CAR T-cells persistence. **A** Clinical outcomes of patients treated with CD19/CD22 CAR T-cells. Clinical outcomes, treatment response of each patient after CD19/CD22 CAR T-cell therapy and the duration of response. Patient number is shown to the left. **B**–**C** Survival analysis. OS and LFS from the day of CAR T-cells infusion are shown for patients who received FC lymphodepletion with DAC (DAC, n = 14) compared with those who received FC alone (CON, n = 12). **D**–**E** Survival analysis of 4 subgroups. The OS and LFS were prolonged by allo-HSCT in all subgroups. **F** The expansion and persistence of CAR T-cells. The presence of CD19/CD22 CAR T-cells in the peripheral blood as assessed by quantitative real-time polymerase chain reaction (PCR) assay. Time points after a second CAR T-cells infusion or allo-HSCT are excluded. We analyzed the kinetics of CAR T-cell counts by using LOESS (Local Polynomial Regression) curve fitting. Bold line represents the averaged data using LOESS curve fitting approximation with the standard error in grey

With a median follow-up of 13 months, there were significant differences in overall survival (OS) and leukemia-free survival (LFS) between both groups (Fig. 1B, C). Patients who underwent allo-HSCT after CAR T-cell therapy in the DAC or the CON group had higher OS and LFS than those without allo-HSCT, although the number of patients in some subgroups was relatively small (Fig. 1D-E). CAR T- cells copies in the peripheral blood were detected by qPCR at several indicated time points after infusion in all patients (Fig. 1F).

There was no statistically significant difference in the incidence of cytokine release syndrome between both groups (Additional file 4: Table S2). All adverse events were reversible and manageable. The medians of peak type 1 helper T (Th1) cytokines (IL-2 and IFN-γ) concentrations were higher in the DAC group than in the CON group. However, in regard to type 2 helper T (Th2) cytokines, the peak value of serum IL-4 after CAR T-cells infusion was significantly higher in the CON group than in the DAC group (P = 0.029; Additional file 5: Table S3).

There are several potential mechanisms underlying the therapeutic benefit of DAC-based lymphodepletion prior to CAR T-cell therapy: 1. DAC can upregulate CD19 expression to make leukemia cells more susceptible to CAR T-cell therapy [5]. 2. DAC can inhibit the methylation of tumor suppressor genes associated with B-ALL and induce leukemia cell apoptosis at high doses. 3. DAC pretreatment can modify the immunosuppressive tumor microenvironment to enhance CAR T-cell efficacy and endogenous immunity, leading to long-term antileukemia immunity [9]. In our study, an increased level of IL-4 was detected in the CON group compared with the DAC group, which suggested that DAC depolarized Th2 cells and inhibited tumor growth [10].

In summary, our data demonstrated that the combination of DAC and FC as a conditioning regimen was safe and effective for Chinese patients with r/r B-ALL. DAC in combination with the FC lymphodepletion regimen may be a new treatment option that can improve the efficacy of CAR T-cell therapy in r/r B-ALL. Moreover, CD19/CD22 CAR T-cell therapy as a bridge to allo-HSCT could be a promising strategy for r/r B-ALL patients to achieve prolonged OS and LFS. However, due to the small sample size and retrospective nature of this study, large-scale randomized controlled clinical trials should be prospectively conducted to confirm our results. Further studies are warranted to determine the key factors and pathways that underlie the synergistic antitumor effect of DAC and CAR T-cells.

## Supplementary Information

**Additional file 1: Figure S1.** A schematic diagram of patient allocation, selection and exclusion. Patients were enrolled in the phase 1/2 clinical trial of CD19/CD22 CAR T-cell therapy (NCT03614858) from October 2017 to May 2021 at the First Affiliated Hospital of Soochow University. The patients received DAC combined with FC depending on disease characteristics such as TP53 mutation, comorbidities, patients' wishes.

**Additional file 2: Figure S2.** Schematic diagram of anti-CD19/CD22 CAR and the clinical protocol procedures. (A) Schematic diagram of anti-CD19/CD22 CAR. The third-generation CAR used in this study was composed of single-chain variable fragments derived from murine monoclonal antibodies against human CD19 and CD22, 2 costimulatory domains from CD28 and OX40, and the CD3-ζchain as the activation domain. SP, signal peptide; VL, variable L chain; VH, variable H chain; TM, transmembrane. (B) Schematic diagram of study procedures. After providing written informed consent, patients with r/r B-ALL underwent leukapheresis, lymphodepleting chemotherapy: FC (fludarabine 30 mg/m^2^/day and cyclophosphamide 300 mg/m^2^/day) on days -5 to -3, with or without DAC (total dose 100 mg/m^2^ in 3 days from day -6 to -4). CD19/CD22 CAR T-cells were infused on successive days from day zero. Bone marrow aspiration was performed for response assessment every month for half a year and every 3 months thereafter. All patients were followed up until they died, lost to follow-up, or withdrew consent. Suitable patients received allo-HSCT within 3 months after CAR T-cell therapy.

**Additional file 3.** Clinical Response.

**Additional file 4.** Adverse Events.

**Additional file 5.** Peak numbers of CAR T-cells and cytokine levels.

## Data Availability

The datasets supporting the conclusions are included within this article.
