# Supplementary figures and images for: Decitabine in combination with fludarabine and cyclophosphamide as a lymphodepletion regimen followed by CD19/CD22 bispecific targeted CAR T-cell therapy significantly improves survival in relapsed/refractory B-ALL patients
Source: Exp Hematol Oncol. 2023 Apr 10;12:36. doi: 10.1186/s40164-023-00397-z (PMC10084593; doi:10.1186/s40164-023-00397-z)

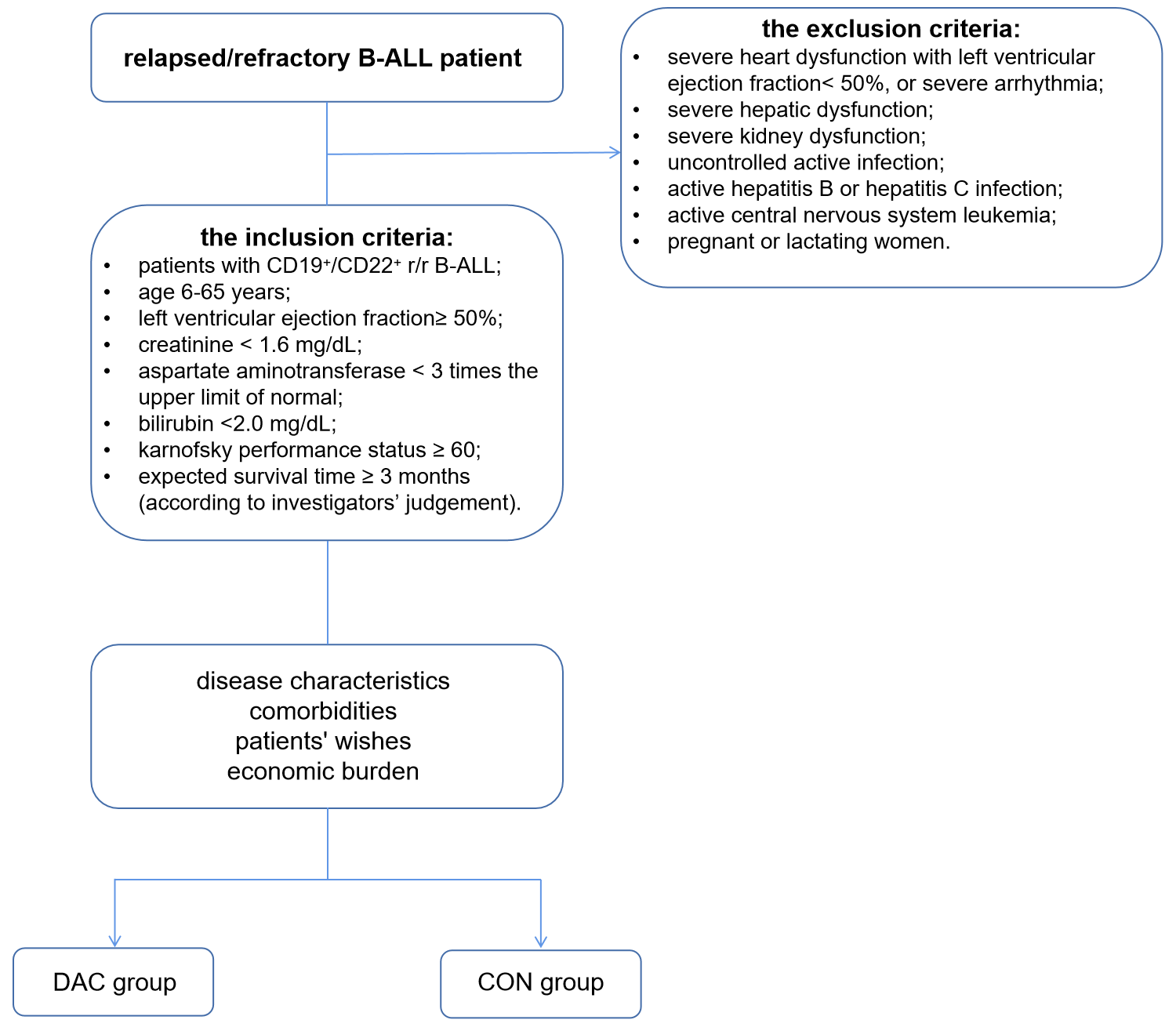

Supplement: Supplementary file 1 — Additional file 1: Figure S1. A schematic diagram of patient allocation, selection and exclusion. Patients were enrolled in the phase 1/2 clinical trial of CD19/CD22 CAR T-cell therapy (NCT03614858) from October 2017 to May 2021 at the First Affiliated Hospital of Soochow University. The patients received DAC combined with FC depending on disease characteristics such as TP53 mutation, comorbidities, patients' wishes. [file 40164_2023_397_MOESM1_ESM.tif]

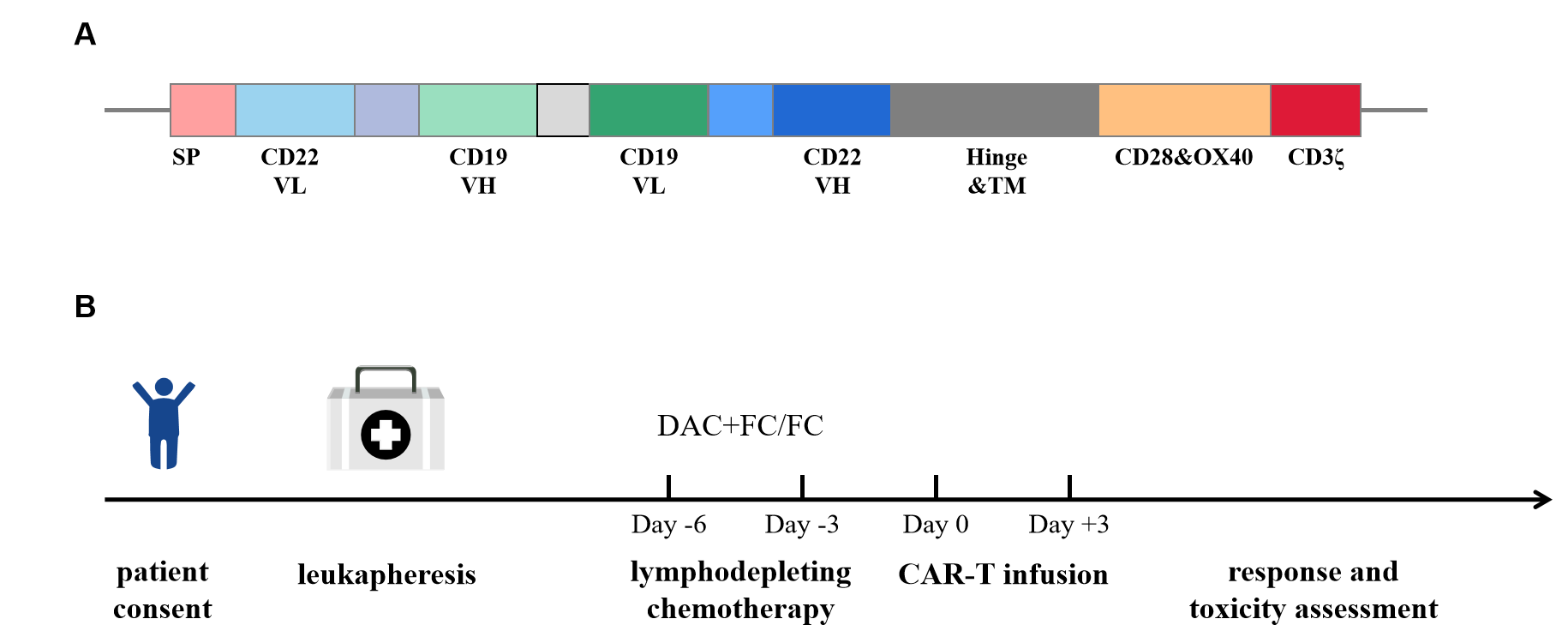

Supplement: Supplementary file 2 — Additional file 2: Figure S2. Schematic diagram of anti-CD19/CD22 CAR and the clinical protocol procedures. (A) Schematic diagram of anti-CD19/CD22 CAR. The third-generation CAR used in this study was composed of single-chain variable fragments derived from murine monoclonal antibodies against human CD19 and CD22, 2 costimulatory domains from CD28 and OX40, and the CD3-ζchain as the activation domain. SP, signal peptide; VL, variable L chain; VH, variable H chain; TM, transmembrane. (B) Schematic diagram of study procedures. After providing written informed consent, patients with r/r B-ALL underwent leukapheresis, lymphodepleting chemotherapy: FC (fludarabine 30 mg/m2/day and cyclophosphamide 300 mg/m2/day) on days -5 to -3, with or without DAC (total dose 100 mg/m2 in 3 days from day -6 to -4). CD19/CD22 CAR T-cells were infused on successive days from day zero. Bone marrow aspiration was performed for response assessment every month for half a year and every 3 months thereafter. All patients were followed up until they died, lost to follow-up, or withdrew consent. Suitable patients received allo-HSCT within 3 months after CAR T-cell therapy. [file 40164_2023_397_MOESM2_ESM.tif]
